# DiB-splits: nature-guided design of a novel fluorescent labeling split system

**DOI:** 10.1038/s41598-020-67095-2

**Published:** 2020-07-06

**Authors:** Nina G. Bozhanova, Alexey S. Gavrikov, Alexander S. Mishin, Jens Meiler

**Affiliations:** 1 Department of Chemistry, Center for Structural Biology, Vanderbilt University, Nashville, TN 37235 USA; 2 Shemyakin-Ovchinnikov Institute of Bioorganic Chemistry, Russian Academy of Sciences, Moscow, 117997 Russia; 3 Institute for Drug Discovery, Leipzig University, Leipzig, SAC 04103 Germany

**Keywords:** X-ray crystallography, Protein design, Biochemistry, Structural biology

## Abstract

Fluorogen-activating proteins (FAPs) are innovative fluorescent probes combining advantages of genetically-encoded proteins such as green fluorescent protein and externally added fluorogens that allow for highly tunable and on demand fluorescent signaling. Previously, a panel of green- and red-emitting FAPs has been created from bacterial lipocalin Blc (named DiBs). Here we present a rational design as well as functional and structural characterization of the first self-assembling FAP split system, DiB-splits. This new system decreases the size of the FAP label to ~8–12 kDa while preserving DiBs’ unique properties: strong increase in fluorescence intensity of the chromophore upon binding, binding affinities to the chromophore in nanomolar to low micromolar range, and high photostability of the protein-ligand complex. These properties allow for use of DiB-splits for wide-field, confocal, and super-resolution fluorescence microscopy. DiB-splits also represent an attractive starting point for further design of a protein-protein interaction detection system as well as novel FAP-based sensors.

## Introduction

Split proteins are engineered proteins which can be reconstituted from two or more parts via non-covalent interactions. The central idea is that reconstitution is followed by regain of a specific function which is abolished in the separated parts. Theoretically, many proteins can be divided into such fragments. In practice, the identification of a functional split protein is still nontrivial, although some success in direct evolution-based^1^, as well as computational^2,3^ design of split systems has been recently demonstrated.

Split proteins were first employed when using ubiquitin for *in vivo* protein-protein interaction detection^4^. Successful cleavage of the reporter protein, dihydrofolate reductase, fused to the C-terminal fragment of ubiquitin was happening only when both the C-terminal and mutated N-terminal fragments of ubiquitin were expressed as fusions to a leucine zipper homodimerization domain but not when expressed individually.

Later, this concept was applied to a number of other proteins. Many of them were enzymes like dihydrofolate reductase^5^, ß-lactamase^6^, thymidine kinase^7^, or luciferase^8^. This allows for real time and quantitative analysis of protein interactions *in vitro* as well as in model organisms. The desire for more user-friendly methods for detecting protein-protein interactions in complex environments and for identification of their precise cellular localization in combination with enormous progress in fluorescent microscopy techniques prompted the creation of fluorescent split proteins. This included split versions of green fluorescent protein (GFP)^9,10^, its differently colored derivatives and homologs^11–13^, far-red emitting phytochrome-based fluorescent proteins^14^, or even dual split reporters^15^.

When used for protein-protein interaction detection, spontaneous self-association of split proteins is highly undesirable. Such self-association events will contribute to the false positive signal and decrease the overall sensitivity of the method. However, spontaneously self-complementing fluorescent split pairs were found to be useful. Their usage allows for substantial decrease of the tag size that is required to be fused to the protein of interest. Therefore, it diminishes potential influence of the tag on the protein of interest behavior^12^.

Fluorogen-activating proteins (FAPs) are a group of unrelated proteins capable of binding to non-protein ligands (fluorogens) and increase the fluorescence quantum yield and/or change spectral properties of these ligands. Some of these FAPs like miniSOG^16^, IFP1.4^17^, iRFP^18^, and UnaG^19^ find their ligands (flavin mononucleotide, biliverdin, and bilirubin) readily available in mammalian cells. Other FAPs like various dye-binding antibodies^20,21^, FAST^22^, DiBs^23^, and *de novo* computationally designed mFAPs^24^ require an exogenous supply of the chromophore. The latter group of FAPs provides multiple benefits. First, available synthetic molecules show a wide range of chemical and photophysical properties allowing for the creation of fluorescent probes with a desired combination of characteristics. Second, external addition of the ligand gives control over the timing and intensity of the fluorescent signal. Third, the noncovalent nature of interaction provides, in some systems, millisecond-scale blinking of the fluorescent signal caused by ligand binding-dissociation events. In these cases, the optimal signal density for high-resolution image reconstruction can be achieved simply by using an appropriate dye concentration. This approach circumvents usage of damaging levels of illumination intensities, which is common for many single-molecule localization microscopy techniques^25^.

Following the extension of the list of reported FAP systems, FAPs-based splits, also known as bimolecular fluorescence complementation (BiFC) systems, begin to appear. That includes multiple bacteriophytochromes-based irreversible^14^ and reversible^1,26^ split systems, photoactive yellow protein-based splitFAST^27^, a label for correlative light and electron microscopy split-miniSOG^28^, and a bilirubin-binding UnaG-based split reporter uPPI^29^. Two of these FAP-based splits, IFP PCA^26^ and splitFAST^27^, require exogenous supply of the chromophore for imaging in eukaryotic cells. It also has been shown that the full-length UnaG fluorescence recovery under photobleaching conditions needs excess bilirubin added into the solution^30^. While IFP chromophore, biliverdin, forms a covalent adduct with the protein, only splitFast and uPPI seem to be suitable for use with binding and dissociation events-detecting single-molecule localization microscopy techniques like protein-PAINT^23^. However, to the best of our knowledge, such an application has not been shown yet for either probe. The reported usage of split-miniSOG for imaging at subdiffraction resolution via electron microscopy is limited to fixed samples.

In our previous work, using a combination of computational and *in vitro* screenings we created a panel of FAPs from bacterial lipocalin Blc (named DiBs) capable of recovering the fluorescence of synthetic analogs of green and red fluorescent proteins’ chromophores^23,31^. Here we report on DiB-splits, a self-assembling FAP split system which has been inspired by the domain-swapped structure of a full-length DiB protein. This new FAP system reduces the size of the label needed to be conjugated with a protein of interest to ~8–12 kDa and is compatible with wide-field, confocal, and super-resolution fluorescence microscopy. DiB-splits also offer an attractive template for design of a protein-protein interaction detection split system as well as FAP-based sensors.

## Results and discussion

### DiB3 domain-swapped crystal structure

**Figure 1:**
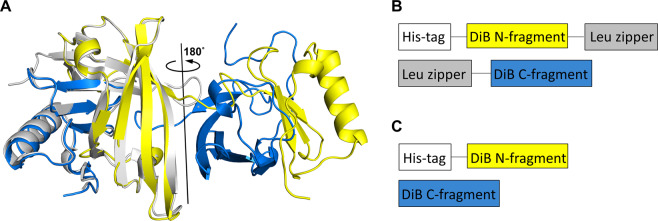
(**A**) Cartoon representation of the domain-swapped DiB3 intertwined dimer (chains colored blue and yellow) overlaid with the structure of the wild type lipocalin Blc (colored light grey, PDB ID 1QWD); Schematic representation of the leucine zippers-containing (“split-Zip”) (**B**) and leucine zippers-free (“split”) (**C**) DiB-split proteins constructs.

The lipocalin fold contains a single eight-stranded continuously hydrogen-bonded antiparallel β-barrel complemented by an α-helix. This common fold has been observed for other lipocalin protein family members^32^, previously characterized wild type Blc (wtBlc) protein^33–35^, as well as another Blc mutant, DiB1, that has been co-crystallized with the M739 (Supplementary Fig. S1)^36^ ligand (Muslinkina *et al*., manuscript submitted). Our attempts to structurally characterize other DiB proteins^23^ in apo and bound states resulted in obtaining protein crystals of DiB3 in the apo form at low pH conditions (pH 3.5) which diffracted to 1.6 Å. The asymmetric unit contains only one protein chain. However, it forms a biological assembly (dimer) with a crystallographic symmetry mate. The intertwined dimer is caused by domain swapping: each of the two Blc-like eight-stranded beta-barrel folds is created by the N-terminus of one of two polypeptide chains and the C-terminus of the other (Fig. 1A). As observed in other domain-swapped structures^37^, the overall lipocalin fold is preserved in the domain-swapped structure (Cα rmsd 1.1 Å), except for the region that connects the exchanging parts of the protein (the hinge region, residues 109–113).

### ***In silico*** analysis

While DiB3 was previously successfully used for *in cellulo* protein labelling^23^, we proposed that the observed domain swapping was driven mainly by the very low pH conditions of the crystallization buffer rather than the private DiB3 mutations (V74F and L141Q). To further evaluate this assumption, we calculated the interaction energies between N- and C-termini fragments of the wtBlc, DiB1, and DiB3 proteins using Rosetta^38^. Despite the fact that wtBlc protein seems to be slightly more stable, DiB3 is not an outlier (Supplementary Fig. S2), suggesting that the introduced mutations are unlikely to cause the domain swapping.

Independent from the reason that caused domain swapping in the crystal, we reasoned that the DiB3 protein as well as other mutants might have two relatively autonomous and stable parts (residues 1 to 108 and 114 to 177). Such property is crucial for the successful creation of a split system. Usually designing a split protein involves laborious screening of multiple protein sites in order to select an appropriate cutting point^11,39,40^. In our case, however, the obtained DiB3 structure is pointing to a potential cleavage site.

### ***In vitro*** evaluation of the proposed split system

 First, we tested whether N- and C-termini fragments, created by separation of a protein chain in the hinge region, are indeed capable to form the lipocalin-like structure when brought together. We fused each of two fragments of DiBs1–3 with one of two leucine zipper peptides as shown on Fig. 1B. We assessed, whether the pairs retained their ability to bind fluorogens and increase their fluorescence brightness if co-expressed in bacteria. For this we added the fluorogen M739 (Supplementary Fig. S1) directly to bacterial suspension, spun down, and examined the pellets under fluorescent microscope. The pellets of all three tested pairs were visibly fluorescent so we proceeded with qualitive assessment (Supplementary Fig. S3) and further with quantitative characterization of the complexes using purified proteins.

### Split-Zip system properties

Split-Zip proteins showed properties similar to the “parental” full-length variants upon addition of the M739 fluorogen including binding affinities, fluorescence spectra maxima, and extinction coefficients (Table 1, Supplementary Figs. S4 and S5). That suggests that when pulled to each other by leucine zippers’ interaction the fragments can successfully restore the lipocalin fold. Interestingly, in two out of three cases (DiB1-split-Zip and DiB2-split-Zip) the apparent binding affinity of the proteins to the ligand slightly increased compared to the corresponding full-length proteins. It might be a result of slowing down the dissociation of the chromophore due to steric hindrance caused by the leucine zippers. Another reason might be a conformational restriction of the residues of the formerly highly flexible loop (residues 109–113)^35^ which locks side chains in the preferable conformation for ligand binding. The opposite effect, weaker binding seen in the case of the red-shifted DiB3-split-Zip:M739 complex, supports the previously suggested hypothesis of the alternative binding mode of the ligand in that complex (Muslinkina *et al*., manuscript submitted).

**Table 1:** Properties of the DiB protein – chromophore M739 complexes.

Name	λ_ex_, nm	λ_em_, nm	*K*_d_, μM	QY, %	ɛ
DiB1	513	541	0.04 ± 0.03	32^a^	49 000
DiB1-split-Zip	517	542	0.023 ± 0.018	31	51 300
DiB1-split	516	543	0.08 ± 0.03	32	50 150
DiB2	507	540	5.9 ± 0.7	37	53 000
DiB2-split-Zip	509	535	4.2 ± 1.2	43	51 300
DiB2-split	509	535	9 ± 0.9	50	49 200
DiB3	545	562	4.9 ± 0.4	15^a^	51 700
DiB3-split-Zip	537	558	8.5 ± 0.8	14	58 000
DiB3-split	533	558	14.1 ± 2.8	16	57 300
M739	517	564	n/a	3.5^a^	53 500^a^

n/a – not available.

λ_ex_ – wavelength of excitation spectrum maximum.

λ_em_ – wavelength of maximum emission intensity.

*K*_d_ values are shown as mean values ± SD of three replicates.

^a^ - data from Bozhanova *et al*. 2017.

The behavior of a split system in the absence of additional “attracting force” like leucine zippers or other interacting proteins determines the range of its possible applications. If the assembly of the split protein is conditional (fails to self-assemble spontaneously), it can be used for investigation of protein-protein interactions^27,41–43^. On the other hand, in the case of spontaneous self-assembly, the split form of the labelling system allows for a reduction in the size of the labelling tag that needs to be added to a protein of interest. Hence, it minimizes tags’ influence on that protein^10,12,44^. Therefore, as a next step we checked the ability of the DiB N- and C-termini fragments to self-assemble. For this we deleted the leucine zippers as shown on Fig. 1C to obtain His-tagged N-termini fragments and untagged C-termini fragments. We co-expressed these new constructs (further referred here as split) and performed immobilized metal ion affinity purification. The affinity tag was present only on the N-termini fragments. Nevertheless, both parts of the split system were co-purified indicating that the assembly occurs spontaneously (Supplementary Figs. S6, S7).

### Split system properties

Self-assembling split proteins retain their ability to bind and increase fluorescence of the fluorogen M739 (Table 1, Supplementary Figs. S4 and S5), although in all three cases we observed a somewhat reduced affinity for M739 by a factor of 2–3. This might be caused by an increased flexibility of the split system causing a higher entropic cost of ligand binding. In all but one case the spectral properties of both DiB-split-Zip and DiB-split systems remained similar to the properties of the full-length protein complex: emission and excitation maxima varied by no more than 5 nm. Only DiB3-split excitation maximum was 12 nm shorter than the one of DiB3 (Supplementary Fig. S4). The reason for this spectral shift has yet to be explored.

### Structural analysis of the DiB2-split protein

**Figure 2:**
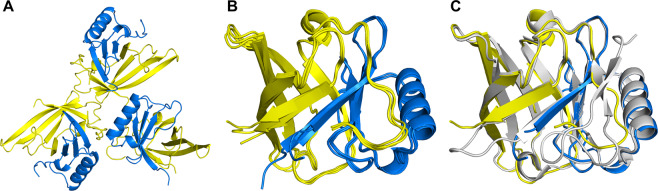
Structural analysis of the DiB2-split protein. Cartoon representation of the (**A**) DiB2-split crystal asymmetric unit, (**B**) overlaid three DiB2-split nonsymmetric monomers, and (**C**) one DiB2-split monomer overlaid with the structure of lipocalin Blc (PDB ID 1QWD, chain (**B**). DiB2-split N-fragments are colored yellow, DiB2-split C-fragments are colored blue, wtBlc is colored light grey.

To further confirm the recovery of the lipocalin fold by split DiBs, we crystalized the DiB2-split protein. Crystals diffracted to approximately 2 Å and contained three “split” molecules per asymmetric unit (Fig. 2A). The oligomeric state of the wtBlc has been previously studied and there are some evidence for its existence as a monomer^35^ as well as a functional dimer^34^. However, according to our knowledge, the possibility of a trimer formation was never investigated before. According to size-exclusion chromatography conducted during purification routine of split proteins there were no signs of oligomerization of any kind (Supplementary Fig. S7). Additionally we used the Protein Interfaces, Surfaces and Assemblies (PISA) server^45^ to assess the biological significance of the observed interfaces between “split” molecules in the trimer. This analysis also suggested that neither of the quaternary structures except for the dimers formed by the N- and C-termini fragments are stable in solution. Thus, we assumed that the observed trimer is solely a result of crystal packing.

The main difference between superimposed monomers from the asymmetric unit is observed in the termini of the two β-strands adjacent to the cleavage site (Fig. 2B). These residues are involved in crystal packing interactions in the DiB2-split crystal. This region, also known as the E/F loop, is capable of adopting multiple conformations even in the full length proteins based on previously obtained structures^35^ (Muslinkina *et al*., manuscript submitted). The residues of the hinge loop and two adjacent β-strands are also responsible for the main difference between wtBlc and DiB2-split monomers (Fig. 2C). The hinge loops of proteins capable of domain swapping are believed to be in an energetically unfavorable conformation in the monomeric state in order to promote domain swapping^37^. The observed differences between structures might be partially caused by releasing the tension in the fold through polypeptide chain cleavage.

Despite these minor structural deviations, the overall lipocalin fold as well as specific intramolecular β-barrel stabilizing interactions, which became intermolecular interactions after the splitting, are well preserved in the DiB2-split structure (Supplementary Fig. S8). This confirms that the split system is capable of spontaneous correct self-assembly to form the functional protein. This finding aligns with the observed photophysical properties.

### ***In vivo*** evaluation of the proposed split system

Out of three DiB-split proteins which we characterized *in vitro*, the DiB2-based split revealed the most favorable properties including high expression levels, stability, and brightness. That is why it was chosen for further evaluation of the DiB-split system for imaging in living cells. For *in vivo* testing we first created two constructs: fusion proteins of the DiB2 fragments with the blue fluorescent protein TagBFP (TagBFP-splitN_1–109_ and TagBFP-splitC_110–177_). We assessed the behavior of these constructs in separate transfections. Cells transfected with the TagBFP-splitC_110–177_ construct alone showed uniform distribution of the blue fluorescence signal throughout cytoplasm and nuclei of the cells (Supplementary Fig. S9B). Separately expressed TagBFP-splitN_1–109_ fusion protein, however, promoted generation of multiple aggregates inside cells (Supplementary Fig. S9A).

### Alternative split point

After inspection of the DiB2-split crystal structure we suggested that the aggregation of the separately expressed splitN_1–109_ fragment might be caused by disturbance of multiple key core interactions caused by removal of the next N-terminus β-strand (Supplementary Fig. S10). We hypothesized that the shift of the split point one β-strand (amino acids 110–125) further to the C terminus of the protein might resolve this problem. We created two new constructs, TagBFP-splitN_1–125_ and the complementary TagBFP-splitC_126–177_, and tested their behavior. As previously, expression of the C terminal part (TagBFP-splitC_126–177_) produced uniformly distributed blue fluorescent signal (Supplementary Fig. S9D). We also discovered significant improvement in behavior of the separately expressed TagBFP-splitN_1–125_ fusion protein. Only cells with very high level of expression showed some residual signs of aggregation (Supplementary Fig. S9C).

### Widefield imaging

Next, we tested the ability of the split system to function *in vivo*. We examined two pairs of available constructs which can form a full-length protein structure: (1) TagBFP-splitN_1–125_ + TagBFP-splitC_126–177_ and (2) TagBFP-splitN_1–125_ + TagBFP-splitC_110–177_. Upon addition of the M739 chromophore, recovery of the DiB2-specific fluorescent signal in green channel was observed only in the second pair (Supplementary Fig. S11). We speculate that this can be caused by somehow compromised integrity of the new splitN_1–125_ fragment. For example, because of presence of an alternative conformation of the new C-terminus. Combination of two protein fragments with partial sequence overlap seems to allow for more efficient assembly or/and longer half-time of the functional complex.

**Figure 3:**
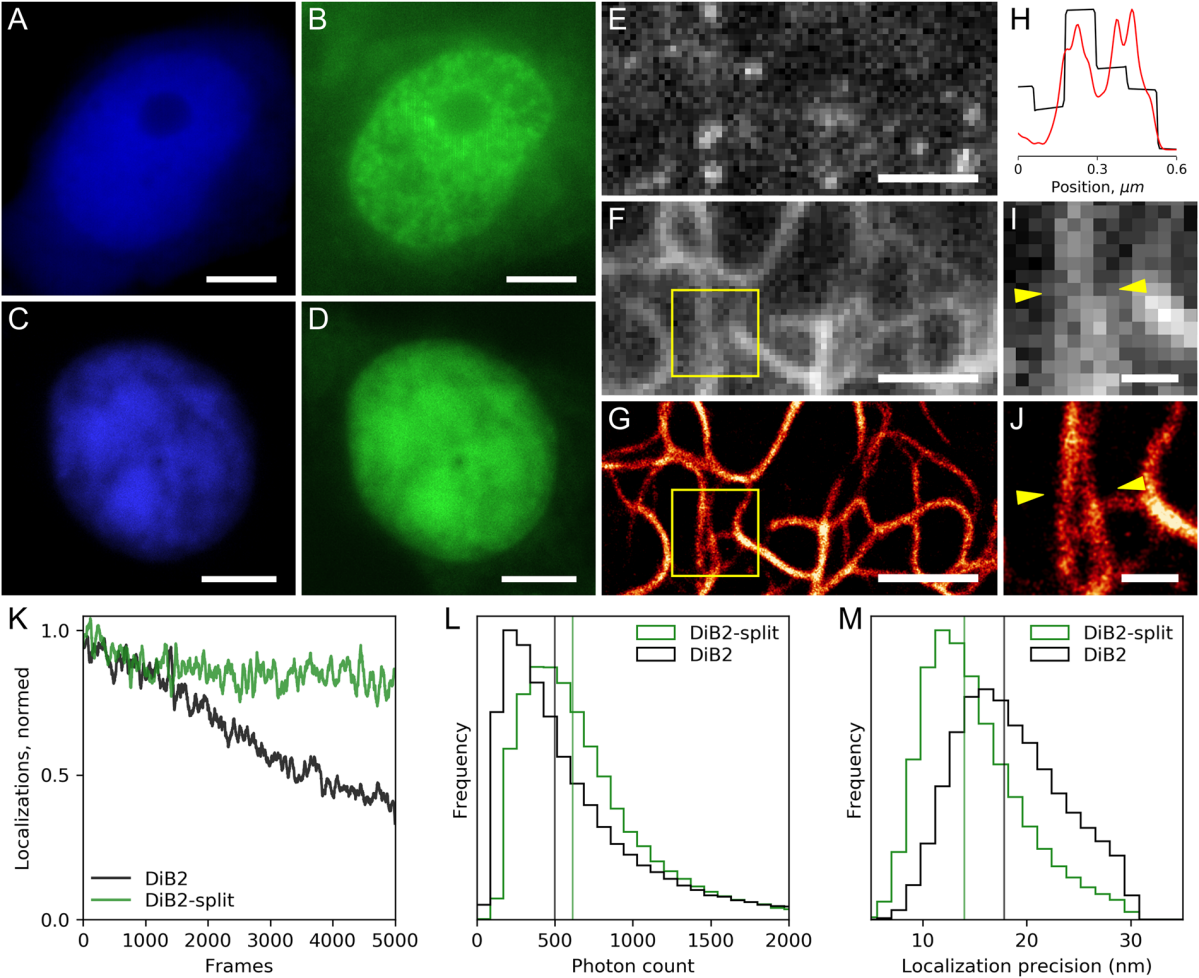
DiB2-split performance in widefield and super-resolution microscopy in living cells. Widefield fluorescence images of HEK293 cells transiently cotransfected with H2B-splitN_1–125_ +TagBFP-splitC_110–177_ (**A**,**B**), and H2B-splitC_110–177_ + TagBFP-splitN_1–125_ (**C**,**D**) constructs in the presence of 200 nM M739; scale bars are 5 µm. Individual frame of a super-resolution series (**E**), average projection of 2 000 frames (**F**) and super-resolution reconstruction (**G**) from 10 000 frames of HeLa Kyoto cell transiently cotransfected with vimentin-splitN_1–125_ + splitC_110–177_-TagBFP constructs in the presence of 25 nM M739; scale bars are 2 μm. (**H**) Normalized intensity profiles between yellow arrows shown on the widefield (**I**) and super-resolution (**J**) regions of images (**F**) and (**G**); black curve – widefield, red curve – super-resolution; scale bars are 500 nm. Comparison of DiB2-split and full-length DiB2 performance in localization microscopy setup (acquisition speed 30 Hz, 1.1 kW cm^−2^ of 488 nm laser) (**K–M**) using vimentin fusion proteins (vimentin-splitN_1–125_ + splitC_110–177_-TagBFP cotransfection and vimentin-DiB2 transfection, correspondingly). (**K**) Comparison of photostability; the graph shows the normalized number of localizations per frame. (**L**) The histogram of detected photons per single molecule event of fluorescence; vertical lines represent the median. (**M**) The histogram of localization precision per single molecule event of fluorescence; vertical lines represent the median.

We further assessed the performance of DiB2-split self-assemblies in living cells by transient cotransfection of splitN_1–125_ and splitC_110–177_ fragments in fusion with either histone H2B or vimentin, and conventional fluorescent protein TagBFP. The assembled split would be visible in a distinct localization in green detection channel if self-assembly occurs successfully. The blue detection channel would show the overall distribution of one of the split halves within a cell and allow for detection of aggregation, non-assembled portion, or other undesirable behavior. We first tested two pairs of H2B-fused proteins: (1) splitN_1–125_ fragment fused with H2B and splitC_110–177_ fused with TagBFP (H2B-splitN_1–125_ + TagBFP-splitC_110–177_), and (2) splitN_1–125_ fragment fused with TagBFP and splitC_110–177_ fused with H2B (H2B-splitC_110–177_ + TagBFP-splitN_1–125_). Upon transient transfection of the first pair of fusion proteins (H2B-splitN_1–125_ + TagBFP-splitC_110–177_), the blue fluorescent signal of the TagBFP confirmed absence of aggregation both in the nucleus and in the cytoplasm (Fig. 3A). In the green detection channel, the DiB2-split:M739 complex signal was visible predominantly in the nucleus indicating effective attraction of the C-terminal part of the split to the nuclei and successful self-assembly of the split system (Fig. 3B). Similar results were obtained with the second set of fusion proteins (H2B-splitC_110–177_ + TagBFP-splitN_1–125_, Fig. 3C,D). In case of vimentin fusion, the fluorescence signal from assembled DiB2-split colocalized with the signal of TagBFP-labeled C-terminal part of the split (Fig. S12). Therefore, we conclude that considerable fraction of the DiB2-split self-assembles spontaneously in living cells from its freely diffusing halves.

### Super-resolution imaging

We further assessed the performance of the DiB2-split system in single-molecule localization super-resolution imaging setup. Similarly to DiB2 (Supplementary video 1), DiB2-split can be used as a protein-PAINT tag: bursts of fluorescence from individual protein-ligand interactions are clearly detectable (Fig. 3E, Supplementary video 2). Super-resolution reconstruction (Fig. 3G) of labeled vimentin shows compatibility of the DiB2-split with this imaging technique and clear resolution improvement over the widefield image (Fig. 3F–J). DiB2-split exhibited higher single-molecule brightness than that of DiB2 (median photon counts per single-molecule event equal to 614 and 501, respectively, Fig. 3L), which ensures super-resolution in live cells with stable number of localizations per frame (Fig. 3K) and higher localization precision (14 nm vs 17.8 nm for DiB2, Fig. 3M). Details of vimentin structure as small as ~30 nm were resolved in live-cell protein-PAINT with either DiB2 or DiB2-split (Supplementary Fig. S13) in short (~1 min) acquisitions.

## Conclusions and future directions

In this study, inspired by the obtained domain-swapped crystal structure of the DiB3 protein in its apo state, we designed and characterized a novel DiB-split FAP system. The two fragments of the split proteins were able to spontaneously reassemble fully restoring fluorogen-activating and spectral properties of the “parental” full-length DiBs. Crystallization of one of these proteins, DiB2-split, further corroborate the preservation of the lipocalin fold by the system. The DiB2-split was tested *in vivo* and was found to give bright and specific fluorescent signal indistinguishable from the one of the full-length protein.

This DiB-split system presents a proof-of-principle demonstration of the potential of the lipocalin scaffold to create a split system. It is immediately applicable as a protein-PAINT^23^ label of a smaller size for super-resolution imaging in living cells. The decrease of the tag size provided through the split can diminish influence of the tag on the protein of interest. *In vitro* data suggest near complete assembly of DiB2 from split fragments, making DiB-splits a feasible replacement for full-length DiBs or fluorescent proteins in cases where the size of the molecular tag matters. Moreover, while DiB-split fluorescence does not require post-assembly chromophore maturation unlike self-assembling fluorescent proteins^10,12^, it can be used for immediate detection of different biological processes such as protein expression and early trafficking events or as a faster reporter of protein solubility.

DiB-splits as well as the parental full-length DiBs have lower signal-to-noise ratio in comparison with FAST family probes. However, FAST localization density decreased rapidly during data acquisition time in single-molecule localization microscopy regime and successful super-resolution imaging using FAST required protocol modifications and usage of oxygen scavengers^46^. Both systems do not require oxygen for their function and might be used in oxygen-deficient systems as it was previously shown with FAST^47^.

DiB-splits would benefit from further optimization of the location of the split point. The original N-terminus fragment of the system (splitN_1–109_) as well as the elongated one (splitN_1–125_) can be redesigned for better stability. Moreover, mutagenesis of the new N- and C-termini could increase binding affinities for the fluorogen. In addition, there is potential for the design of a variety of other fluorescent tools. For example, redesign of the intramolecular interface of the DiB-split proteins reported here to promote higher independence of the N- and C-termini fragments can result in a new FAP-based tool for protein-protein interactions detection. Such tool would complement the existing mEos3.2^48^ and PAmCherry1-based^49^ super-resolution imaging compatible BiFC labels providing additional benefit of fast and oxygen level independent measurements. Spatial proximity of the natural N- and C-termini of the protein makes DiB proteins a promising starting point for the design of DiB-based circularly permuted proteins. While the discovered split point is close to the ligand binding site, such circularly permuted proteins represent a promising starting point for DiB-based biosensors design. Successful permutation might also allow for the creation of a new self-assembling split system with smaller parts analogous to self-complementing split fluorescent GFP11^10^ and sfCherry11^12^ tags.

## Methods

### Molecular cloning

Plasmids pMRBad-Z-CspGFP (Addgene plasmid #40730) and pET11a-Z-NspGFP (Addgene plasmid #40729)^50^ were a gift from Brian McNaughton and were used to create Blc-split-Zip vectors. First, we amplified N-fragments (residues 11 to 109) and C-fragments (residues 110 to 177) of the DiB mutants^23^ as well as leucine zipper peptides with adjacent upstream or downstream portions of the vector from the pMRBad-Z-CspGFP and pET11a-Z-NspGFP plasmids, correspondingly, and the upstream portion of the pET11a-Z-NspGFP plasmid including His-tag. Second, we used the overlap PCR to create DNA fragments containing leucine zipper peptides fused with N- or C-fragments of the DiB mutants flanked by upstream and downstream portions of the vector. These fragments were digested with *BamHI* and *XbaI* or *NcoI* and *BsrGI* restriction enzymes and ligated in the original vectors. These vectors were further used to create split fragments without leucine zipper peptides. For this we amplified N-fragments of the DiB mutants with adjacent upstream portion of the vector introducing stop codon and *BamHI* restriction site instead of leucine zipper peptide coding sequence and C-fragments of the DiB mutants introducing start codon and *NcoI* restriction site instead of leucine zipper peptide. The PCR products were again digested with *BamHI* and *XbaI* or *NcoI* and *BsrGI* restriction enzymes and ligated in the original vectors.

DiB2-split fusions with H2B, vimentin, and TagBFP were generated by Golden Gate Assembly^51–53^. The resulted constructs’ amino acid sequences are provided below. The linker sequences are underlined.

>H2B-splitN_1–125_

MPEPAKSAPAPKKGSKKAVTKAQKKGGKKRKRSRKESYSIYVYKVLKQVHPDTGISSKAMGIMNSFVNDIFERIAGEASRLAHYNKRSTITSREIQTAVRLLLPGELAKHAVSEGTKAITKYTSAKDPPVATMASSPTPPRGVTVVNNFDCKRYLGTWYEIARFDHRFERGLEKVTATYSLRDDGGLNVINKGYNPDRGMWQQSEGKAYFTGAPTRAALKVSFFGPFYGGYNVIALDREYSG*

>TagBFP-splitC_110–177_

MSELIKENMHMKLYMEGTVDNHHFKCTSEGEGKPYEGTQTMRIKVVEGGPLPFAFDILATSFLYGSKTFINHTQGIPDFFKQSFPEGFTWERVTTYEDGGVLTATQDTSLQDGCLIYNVKIRGVNFTSNGPVMQKKTLGWEAFTETLYPADGGLEGRNDMALKLVGGSHLIANIKTTYRSKKPAKNLKMPGVYYVDYRLERIKEANNETYVEQHEVAVARYCDLPSKLGHKLNDPPVATMGPFYGGYNVIALDREYRHALVCGPDRDYLWILSRTPTISDEVKQEMLAVATREGFDVSKFIWVQQPGSG*

>H2B-splitC_110–177_

MPEPAKSAPAPKKGSKKAVTKAQKKGGKKRKRSRKESYSIYVYKVLKQVHPDTGISSKAMGIMNSFVNDIFERIAGEASRLAHYNKRSTITSREIQTAVRLLLPGELAKHAVSEGTKAITKYTSAKDPPVATMGPFYGGYNVIALDREYRHALVCGPDRDYLWILSRTPTISDEVKQEMLAVATREGFDVSKFIWVQQPGSG*

>TagBFP-splitN_1–125_

MSELIKENMHMKLYMEGTVDNHHFKCTSEGEGKPYEGTQTMRIKVVEGGPLPFAFDILATSFLYGSKTFINHTQGIPDFFKQSFPEGFTWERVTTYEDGGVLTATQDTSLQDGCLIYNVKIRGVNFTSNGPVMQKKTLGWEAFTETLYPADGGLEGRNDMALKLVGGSHLIANIKTTYRSKKPAKNLKMPGVYYVDYRLERIKEANNETYVEQHEVAVARYCDLPSKLGHKLNDPPVATMASSPTPPRGVTVVNNFDCKRYLGTWYEIARFDHRFERGLEKVTATYSLRDDGGLNVINKGYNPDRGMWQQSEGKAYFTGAPTRAALKVSFFGPFYGGYNVIALDREYSG*

>vimentin-splitN_1–125_

MSTRSVSSSSYRRMFGGPGTASRPSSSRSYVTTSTRTYSLGSALRPSTSRSLYASSPGGVYATRSSAVRLRSSVPGVRLLQDSVDFSLADAINTEFKNTRTNEKVELQELNDRFANYIDKVRFLEQQNKILLAELEQLKGQGKSRLGDLYEEEMRELRRQVDQLTNDKARVEVERDNLAEDIMRLREKLQEEMLQREEAENTLQSFRQDVDNASLARLDLERKVESLQEEIAFLKKLHEEEIQELQAQIQEQHVQIDVDVSKPDLTAALRDVRQQYESVAAKNLQEAEEWYKSKFADLSEAANRNNDALRQAKQESTEYRRQVQSLTCEVDALKGTNESLERQMREMEENFAVEAANYQDTIGRLQDEIQNMKEEMARHLREYQDLLNVKMALDIEIATYRKLLEGEESRISLPLPNFSSLNLRETNLDSLPLVDTHSKRTLLIKTVETRDGQVINETSQHHDDLEGDPPVATGMASSPTPPRGVTVVNNFDCKRYLGTWYEIARFDHRFERGLEKVTATYSLRDDGGLNVINKGYNPDRGMWQQSEGKAYFTGAPTRAALKVSFFGPFYGGYNVIALDREYSG*

>splitC_110–177_-TagBFP

MGPFYGGYNVIALDREYRHALVCGPDRDYLWILSRTPTISDEVKQEMLAVATREGFDVSKFIWVQQPGSGDPPVATMSELIKENMHMKLYMEGTVDNHHFKCTSEGEGKPYEGTQTMRIKVVEGGPLPFAFDILATSFLYGSKTFINHTQGIPDFFKQSFPEGFTWERVTTYEDGGVLTATQDTSLQDGCLIYNVKIRGVNFTSNGPVMQKKTLGWEAFTETLYPADGGLEGRNDMALKLVGGSHLIANIKTTYRSKKPAKNLKMPGVYYVDYRLERIKEANNETYVEQHEVAVARYCDLPSKLGHKLN*

>TagBFP-splitN_1–109_

MSELIKENMHMKLYMEGTVDNHHFKCTSEGEGKPYEGTQTMRIKVVEGGPLPFAFDILATSFLYGSKTFINHTQGIPDFFKQSFPEGFTWERVTTYEDGGVLTATQDTSLQDGCLIYNVKIRGVNFTSNGPVMQKKTLGWEAFTETLYPADGGLEGRNDMALKLVGGSHLIANIKTTYRSKKPAKNLKMPGVYYVDYRLERIKEANNETYVEQHEVAVARYCDLPSKLGHKLNDPPVATMASSPTPPRGVTVVNNFDCKRYLGTWYEIARFDHRFERGLEKVTATYSLRDDGGLNVINKGYNPDRGMWQQSEGKAYFTGAPTRAALKVSFFSG*

>TagBFP-splitC_126–177_

MSELIKENMHMKLYMEGTVDNHHFKCTSEGEGKPYEGTQTMRIKVVEGGPLPFAFDILATSFLYGSKTFINHTQGIPDFFKQSFPEGFTWERVTTYEDGGVLTATQDTSLQDGCLIYNVKIRGVNFTSNGPVMQKKTLGWEAFTETLYPADGGLEGRNDMALKLVGGSHLIANIKTTYRSKKPAKNLKMPGVYYVDYRLERIKEANNETYVEQHEVAVARYCDLPSKLGHKLNDPPVATMRHALVCGPDRDYLWILSRTPTISDEVKQEMLAVATREGFDVSKFIWVQQPGSG*

>vimentin-DiB2

MSTRSVSSSSYRRMFGGPGTASRPSSSRSYVTTSTRTYSLGSALRPSTSRSLYASSPGGVYATRSSAVRLRSSVPGVRLLQDSVDFSLADAINTEFKNTRTNEKVELQELNDRFANYIDKVRFLEQQNKILLAELEQLKGQGKSRLGDLYEEEMRELRRQVDQLTNDKARVEVERDNLAEDIMRLREKLQEEMLQREEAENTLQSFRQDVDNASLARLDLERKVESLQEEIAFLKKLHEEEIQELQAQIQEQHVQIDVDVSKPDLTAALRDVRQQYESVAAKNLQEAEEWYKSKFADLSEAANRNNDALRQAKQESTEYRRQVQSLTCEVDALKGTNESLERQMREMEENFAVEAANYQDTIGRLQDEIQNMKEEMARHLREYQDLLNVKMALDIEIATYRKLLEGEESRISLPLPNFSSLNLRETNLDSLPLVDTHSKRTLLIKTVETRDGQVINETSQHHDDLEGDPPVATMASSPTPPRGVTVVNNFDCKRYLGTWYEIARFDHRFERGLEKVTATYSLRDDGGLNVINKGYNPDRGMWQQSEGKAYFTGAPTRAALKVSFFGPFYGGYNVIALDREYRHALVCGPDRDYLWILSRTPTISDEVKQEMLAVATREGFDVSKFIWVQQPGSG*

Correctness of all obtained constructs was confirmed by sequencing.

### Protein expression and purification

All proteins were expressed in XJb(DE3) Autolysis (Zymo Research) *E. coli* strain. Cells were grown in LB media supplemented with 100 µg/mL ampicillin (full-length DiB proteins) or 100 µg/mL ampicillin and 50 µg/mL kanamycin (split-Zip and split proteins) at 37 °C. Expression was induced by addition 0.04% L-arabinose (full-length DiB proteins) or 0.2% L-arabinose and 10 μM IPTG (split-Zip and split proteins) at 0.8 OD. Cells were harvested after 3 hours of expression at 37 °C and were resuspended in PBS buffer, pH 7.4. Suspensions were frozen at −80 °C and thawed at room temperature three times. DNA was destroyed by short sonication and the lysates were centrifuged to obtain cell-free extracts. The proteins were first purified using gravity flow columns with TALON metal affinity resin (Clontech) and further purified by size-exclusion chromatography on a HiLoad 16/600 Superdex 75 pg or Superdex 200 pg 10/300 GL column (GE Healthcare) pre-equilibrated with 50 mM sodium phosphate buffer, pH 6.0.

### Protein concentration calculation

Protein concentrations were estimated using the Bradford dye-binding method-based^54^ colorimetric assay (Bio-Rad) and bovine serum albumin standard. Single point absorption measurements (595 nm) were performed using FlexStation 3 microplate reader (Molecular Devices). All measurements were performed in triplicate.

### Fluorescence spectra detection

Horiba Jobin Yvon Fluoromax-3 fluorometer was used to detect full fluorescence excitation and fluorescence emission spectra for excitation/emission maxima evaluation.

### Quantum yield measurements

Fluorescence quantum yield was measured relative to a known standard keeping all instrumental conditions identical. Previously characterized DiB:M739 complexes as well as free M739 chromophore were used as standards. Absorbance spectra were detected using double-beam Shimadzu UV-1800 UV/Vis spectrophotometer. Fluorescence spectra were measured using Horiba Jobin Yvon Fluoromax-3 fluorometer.

### Chromophore binding analysis

Titrations were performed and analyzed as previously described^23^ using FlexStation 3 microplate reader (Molecular Devices). In brief, constant amount of the chromophore solution was added to protein solutions of different concentrations. The full fluorescence emission spectra were collected using wavelength close to protein-chromophore complex excitation spectrum maximum wavelength. Fluorescence intensity at complex emission spectrum maximum wavelength was extracted and used to determine apparent dissociation constants (*K*_d_).

### Crystallization, Data Collection, and Structure Determination

DiB3 (10 mg/mL in 50 mM sodium phosphate buffer, pH 6.0) was crystalized at 21 °C in 0.8 M sodium citrate, 50 mM sodium borate, 0.1 sodium acetate, pH 3.5 with protein to precipitant volume ratio of 1:1 using hanging drop vapor diffusion technique. Crystals grew within 1–3 days and were flash frozen in liquid nitrogen using Parabar 10312 oil as cryoprotectant.

DiB2-split (12 mg/mL in 50 mM sodium phosphate buffer, pH 6.0) crystals were obtained at 21 °C in 1.6 M ammonium sulphate, 0.1 M MES, pH 4.5 with protein to precipitant volume ratio of 1:1 or in 1.6 M ammonium sulphate, 0.1 M MES, pH 4.5 supplemented with 0.1 M Iron(III) chloride hexahydrate or 5% w/v n-Dodecyl-b-D-maltoside according to Hampton Research Additive Screen protocol using sitting drop vapor diffusion technique. Crystals grew within 1 week and were flash frozen in liquid nitrogen using Parabar 10312 oil as cryoprotectant.

Diffraction data were collected at the Life Sciences Collaborative Access Team beamline 21-ID-G at the Advanced Photon Source, Argonne National Laboratory. The diffraction data were processed using xia2 software suite^55^. The crystal structures were solved by molecular replacement with MOLREP^56^ using the wtBlc structure (PDB ID 1QWD) as a search model. Models building and iterative refinement were performed with Coot^57^ and REFMAC^58^, respectively. The final statistics of the structures are shown in Supplementary Table S1. The models have been deposited into the Protein Data Bank (PDB ID 6UKK and 6UKL). Structure figures were prepared using PyMol (v.2.2.3, Schrodinger, LLC).

### Cell culture and transient transfection

HEK293 and HeLa Kyoto cells were grown in Dulbecco’s modification of Eagle’s medium (DMEM) (PanEco) supplied with 50 U/ml penicillin and 50 µg/ml streptomycin (PanEco), 2 mM L-glutamine (PanEco) and 10% fetal bovine serum (HyClone, Thermo Scientific) at 37 °C and 5% CO_2_. For transient transfections FuGENE 6 reagent (Roche) was used. Immediately before imaging DMEM was replaced with HHBS media (Hanks Buffer supplemented with 20 mM Hepes).

### Fluorescence microscopy

Widefield fluorescence microscopy was performed with the Leica DMI6000B inverted microscope, Zyla 5.5 sCMOS camera (Andor), CoolLED pE-300 light source, GFP and BFP filter sets. Single-molecule localization super-resolution imaging of living cells was performed with Nanoimager S (ONI). Imaging in HILO mode was performed with  1.1 kW cm^−2^ of 488 nm laser light intensity. Typical acquisitions were 10 000 frames taken at a frequency of 30 Hz.

### Super-resolution images processing

Localizations during acquisition were detected using NimOS 1.6.1.9898 (ONI, UK). Super-resolution image reconstruction was performed using default values of photon, precision and sigma filters in NimOS. Data analysis was performed using a custom-made Python script. Image resolution was determined by decorrelation analysis plugin^59^.

### Interface energy calculations using Rosetta

To prepare DiB3 structure for analysis we reconstructed the intertwined dimer using crystallographic symmetry and saved the N-terminus portion of one protein chain (residues 24 to 109) and the C-terminus portion of the other protein chain (residues 114 to 175) as a single pdb file. Both wtBlc (PDB ID 1QWD) and DiB1 crystal structures contain two protein chains in the asymmetric unit. We separated the chains into different pdb files and used both for analysis. Each “original” chain was further separated into two chains mimicking DiB3-split and only residues 24 to 109, and 114 to 175 were left to make the length of all structures equal. Each of the 5 prepared structures was then relaxed and the interface energy between two chains was calculated using the following protocol:

<ROSETTASCRIPTS > 

<SCOREFXNS/ > 

<TASKOPERATIONS/ > 

<MOVERS > 

<FastRelax name = “fastrelax” relaxscript = “default”/>

<ClearConstraintsMover name = “clear”/>

<InterfaceAnalyzerMover ligandchain = “B” name = “iface_analyzer” pack_input = “0” pack_separated = “1” packstat = “0” scorefxn = “REF2015” tracer = “0”/>

</MOVERS > 

<FILTERS/ > 

<APPLY_TO_POSE/ > 

<PROTOCOLS > 

<Add mover = “fastrelax”/>

<Add mover = “clear”/>

<Add mover = “iface_analyzer”/>

</PROTOCOLS > 

<OUTPUT scorefxn = “REF2015”/ > 

</ROSETTASCRIPTS > 

The protocol was repeated independently 100 times for each of the starting structures. The data obtained for two separate chains in the asymmetric unit from wtBlc and DiB1 crystal structures were combined. The distribution of the obtained interface energies between N- and C-terminus fragments is shown on Supplementary Fig. S2.

## Supplementary information

Supplementary information.

Video1

Video2

## Data Availability

The crystal structures reported in this paper have been deposited to the Protein Data Bank under accession numbers 6UKK and 6UKL. All other relevant data are included with the manuscript.
