# Supplementary material for: DiB-splits: nature-guided design of a novel fluorescent labeling split system
Source: Sci Rep. 2020 Jul 6;10:11049. doi: 10.1038/s41598-020-67095-2 (PMC7338535; doi:10.1038/s41598-020-67095-2)
Supplement: Supplementary file 1 — Supplementary information. [file 41598_2020_67095_MOESM1_ESM.pdf]

DiB-splits: nature-guided design of a novel fluorescent labeling split system.

Nina G. Bozhanova[1], Alexey S. Gavrikov[2], Alexander S. Mishin[2], Jens Meiler[1,3*]

[1] *Department of Chemistry, Center for Structural Biology, Vanderbilt University, Nashville, TN 37235, USA*

[2] *Shemyakin-Ovchinnikov Institute of Bioorganic Chemistry, Russian Academy of Sciences, Moscow, 117997, Russia*

[3] *Institute for Drug Discovery, Leipzig University, Leipzig, SAC 04103, Germany*

[*]corresponding author: jens.meiler@vanderbilt.edu

Supplementary information

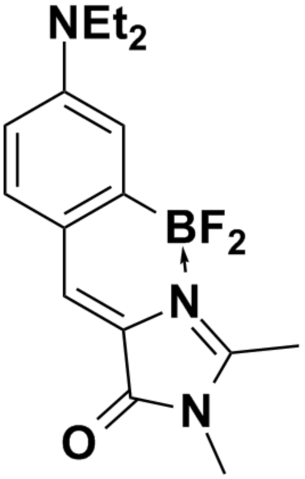

Supplementary Figure S1. M739 chromophore structure.

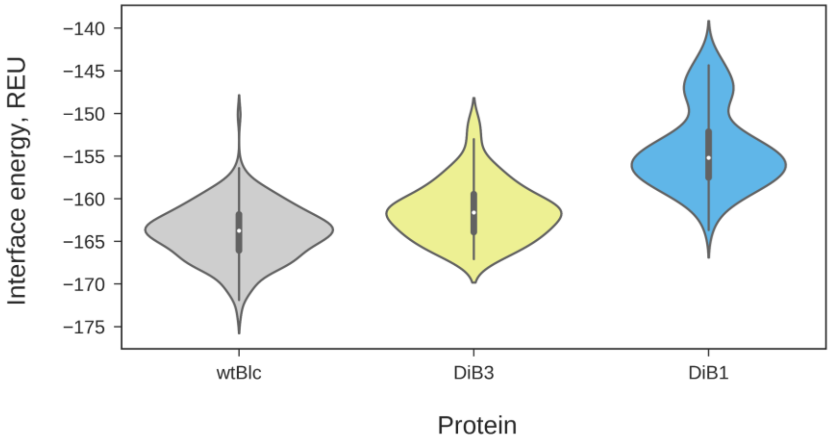

Supplementary Figure S2. Violin plots showing distribution of the interface energies (REU – Rosetta Energy Units) between N- and C-termini fragments of the three evaluated proteins calculated using Rosetta. The median value is shown as a white dot, the central thick bar represents the interquartile range, the thin line represents 1.5x interquartile range.

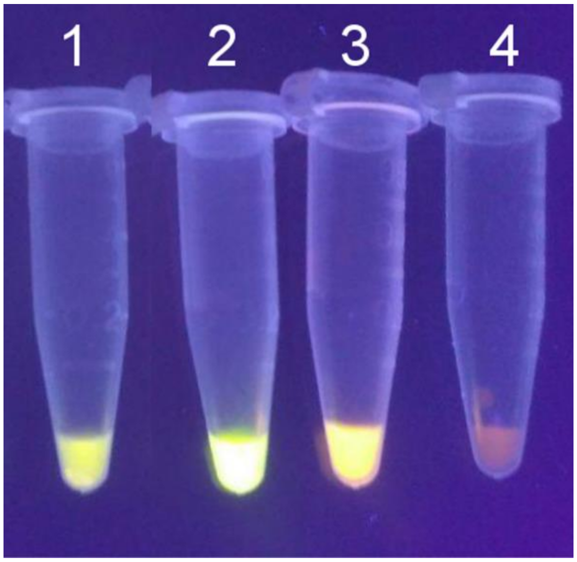

Supplementary Figure S3. Fluorescence of the DiB-split-Zip proteins in presence of M739 (1-3) and free M739 (4) solutions under UV light. (1) DiB2-split-Zip; (2) DiB1-split-Zip; (3) DiB3-split-Zip.

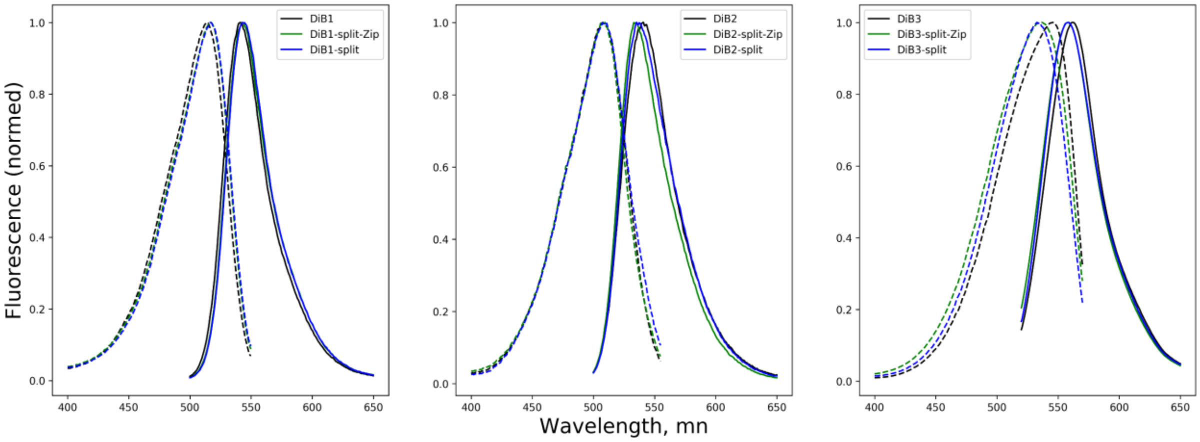

Supplementary Figure S4. Fluorescence emission (solid lines) and fluorescence excitation (dashed lines) spectra of full-length DiB, DiB-split-Zip, and DiB-split proteins in complex with chromophore M739.

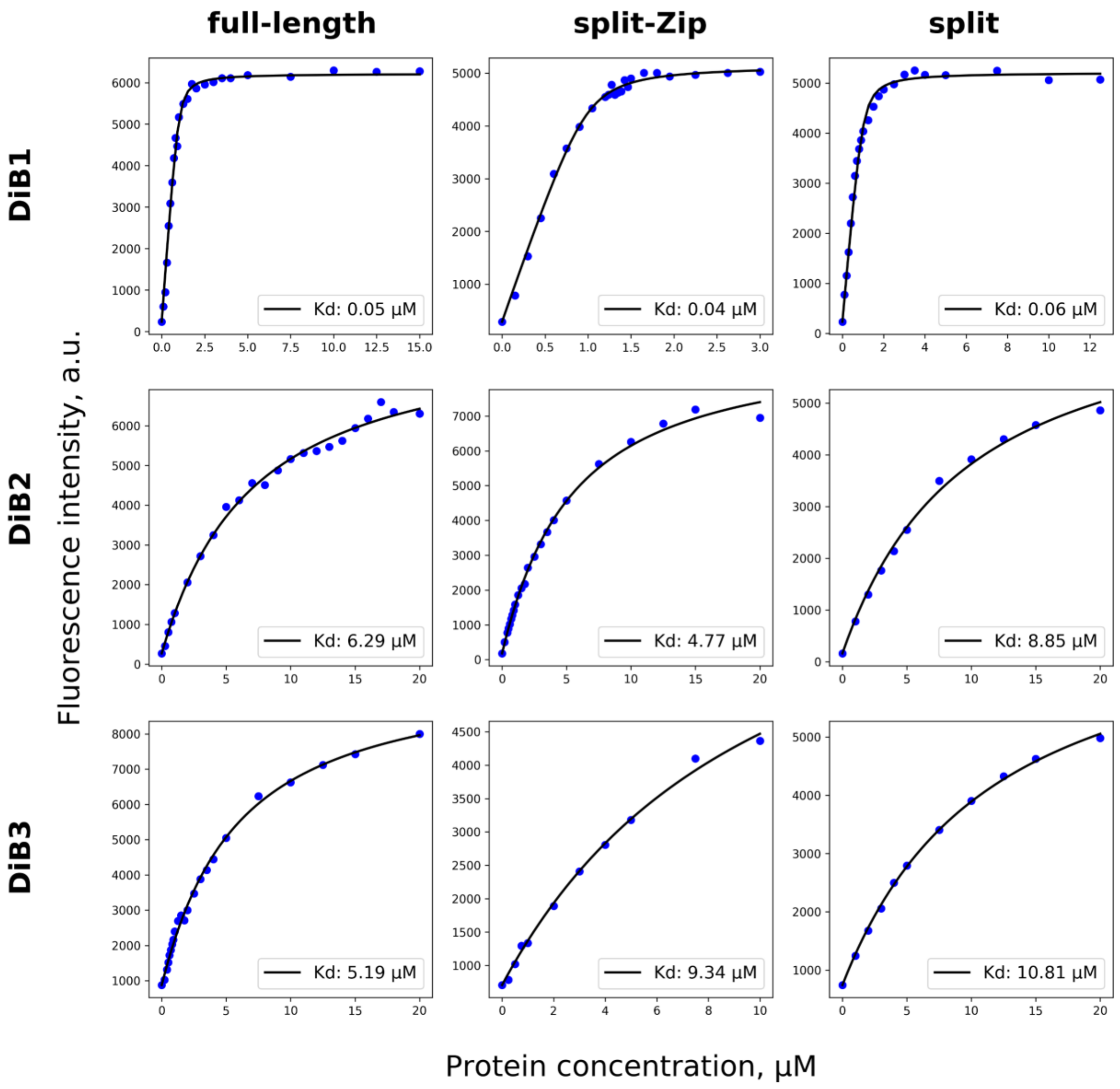

Supplementary Figure S5. Representative examples of full-length DiB, DiB-split-Zip, and DiB-split proteins fluorescence titration with chromophore M739 experiments. Blue dots show measured values, black lines represent fitted curves. Corresponding K_d values are indicated on the plots.

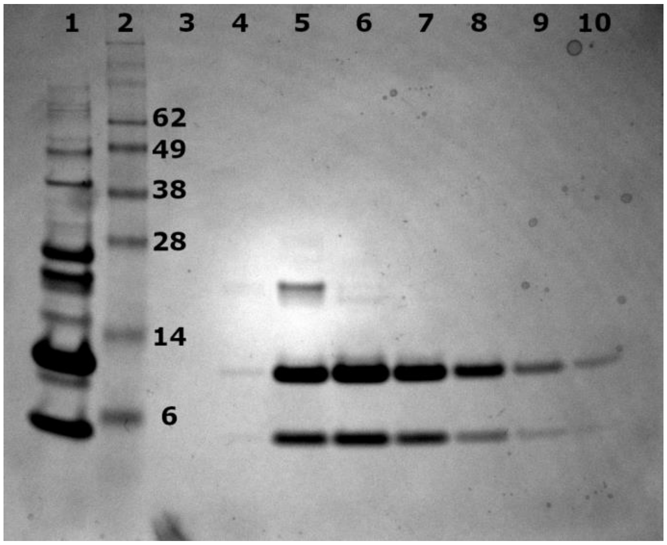

Supplementary Figure S6. Representative SDS-PAGE analysis (10% acrylamide) of a DiB-split protein fragments co-purification. (1) Elution from metal affinity gravity flow column; (2) Molecular weight standard, approximate molecular weights in kDa of the bands are specified on the figure next to the corresponding bands; (3-10) Size-exclusion chromatography fractions from the DiB-split protein peak.

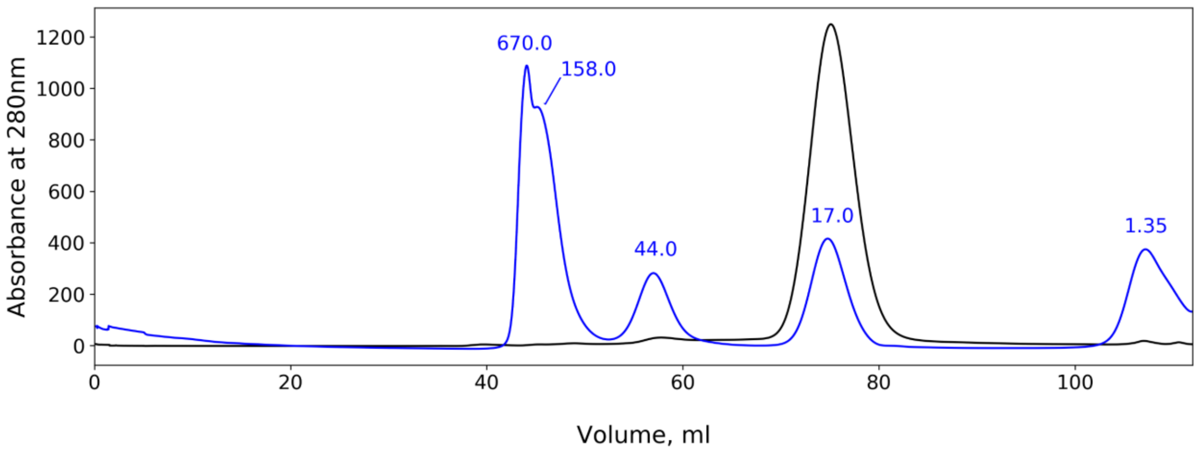

Supplementary Figure S7. Representative preparative size-exclusion chromatogram of a DiB-split protein (black line) and of the Bio-Rad's gel filtration standard (blue line) on a HiLoad 16/600 Superdex 75 pg column in 50 mM sodium phosphate buffer, pH 6.0 at 4 °C. Molecular weights (in kDa) of the standard's components are labeled above the corresponding peaks. Based on the elution volume, DiB-split proteins (~0.5 mM) migrate as expected for ~20 kDa proteins. There are no signs of any significant oligomerization of any kind.

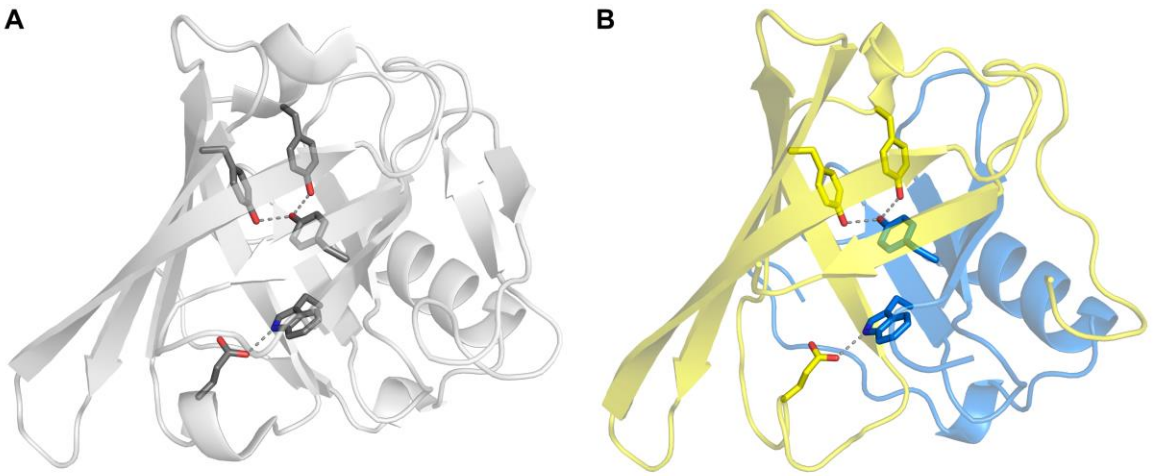

Supplementary Figure S8. Intramolecular β-barrel stabilizing interactions of lipocalin Blc (PDB ID 1QWD, chain B) (A) are preserved in the DiB2-split structure (B). DiB2-split N-fragment is colored yellow, DiB2-split C-fragment is colored blue. Grey dotted lines represent hydrogen bonds between amino acids shown as sticks.

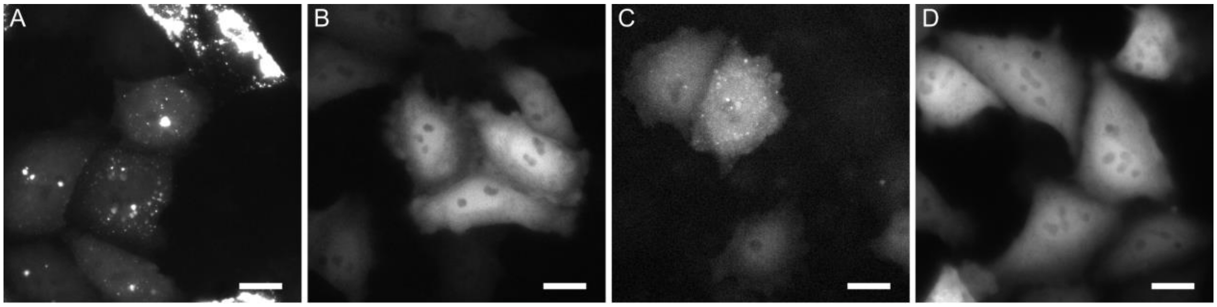

Supplementary Figure S9. Widefield fluorescence images of HEK293 cells transiently transfected with (A) TagBFP-splitN_{1-109}, (B) TagBFP-split$C_{110-177}$, (C) TagBFP-splitN_{1-125}, or (D) TagBFP-split$C_{126-177}$ constructs. Signal from a blue channel. Scale bars are 10 µm.

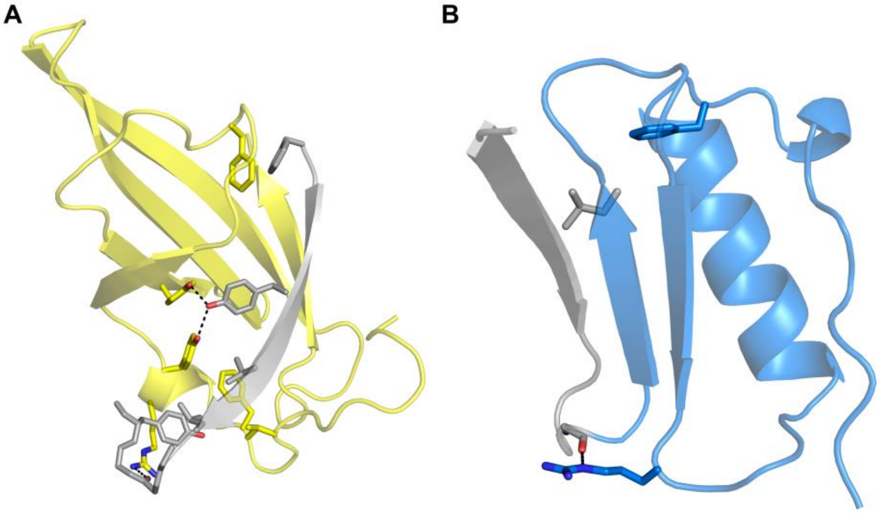

Supplementary Figure S10. Distant interactions of the 16 amino acid long β-strand (shown are amino acids 112 – 125, colored grey) with (A) the N-fragment (colored yellow) and (B) remains of the C-fragment (colored blue) of the protein. Distant interactions in this case are defined as interactions with amino acids outside the adjacent β-strand. Main interacting residues are shown as sticks. Black dotted lines represent hydrogen bonds.

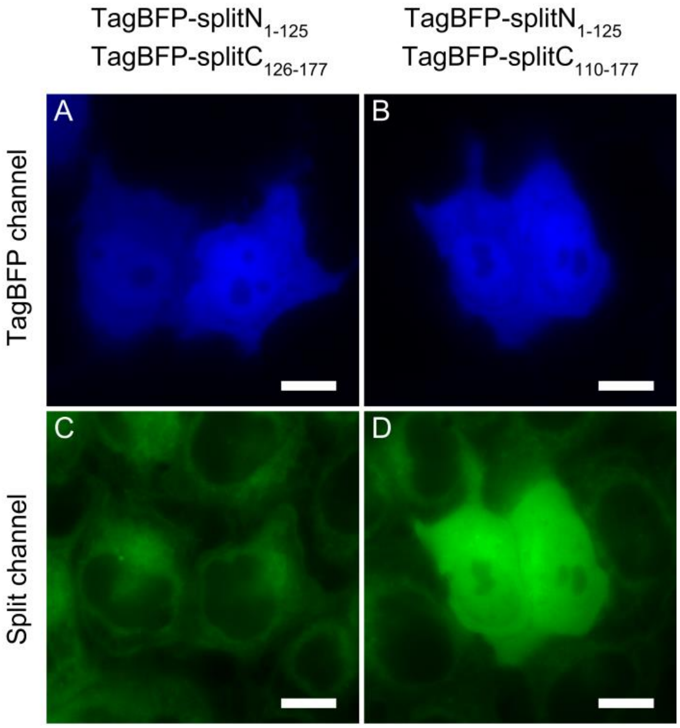

Supplementary Figure S11. Different DiB2-split fragments combinations' performance evaluation. Widefield fluorescence images of HEK293 cells transiently cotransfected with TagBFP-splitN$_{1\text{-}125}$ + TagBFP-splitC$_{126\text{-}177}$ (A, C) or with TagBFP-splitN$_{1\text{-}125}$ + TagBFP-splitC$_{110\text{-}177}$ (B, D) constructs in the presence of 200 nM M739. Scale bars are 10 μm.

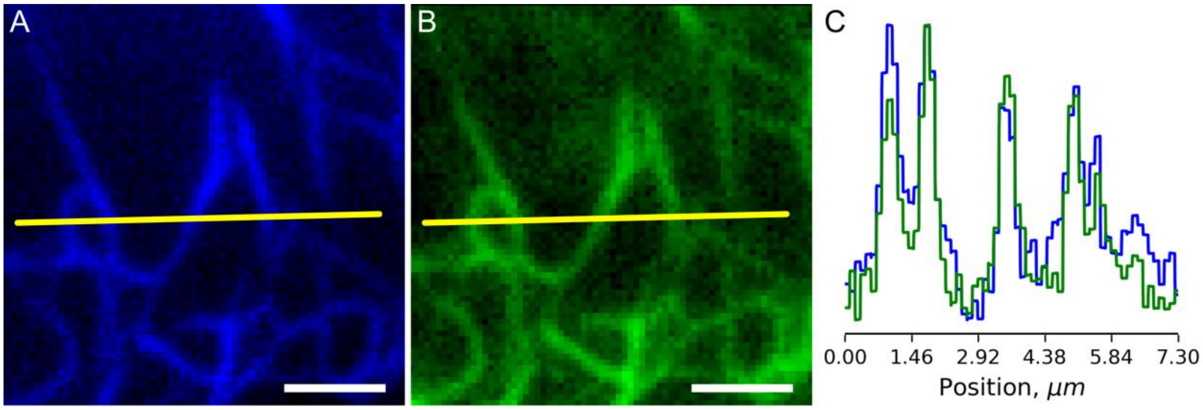

Supplementary Figure S12. Analysis of the efficiency of the DiB2-split protein self-assembly in eukaryotic cells. HeLa Kyoto cells transiently cotransfected with vimentin-splitN$_{1-125}$ + splitC$_{110-177}$-TagBFP constructs in the presence of 25nM M739; scale bars are 2 μm. (A) Widefield image with 405 nm laser excitation (TagBFP channel), (B) Average projection of 2 000 frames of DiB2-split with 488 nm laser excitation. (C) Normalized fluorescence intensity profiles along the yellow lines shown on the images A and B; blue line – TagBFP, green line – DiB2-split.

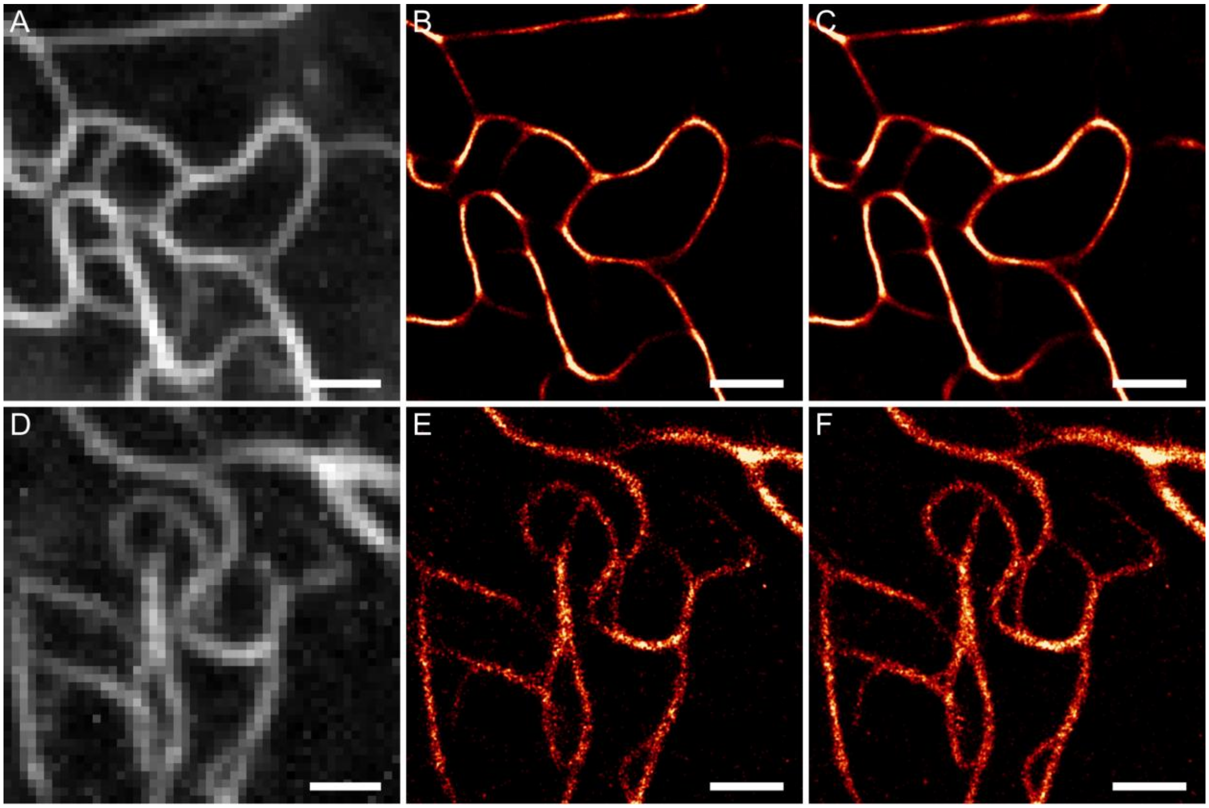

Supplementary Figure S13. Super-resolution imaging of DiB2 and DiB2-split. HeLa Kyoto cells transiently transfected with vimentin-DiB2 (A-C) or vimentin-splitN$_{1-125}$ + splitC$_{110-177}$-TagBFP (D-F) constructs in the presence of 25nM M739. (A, D) Average projections of 2 000 frames of DiB2 and DiB2-split respectively with 488 nm laser excitation. Super-resolution reconstruction from 2 000 frames of DiB2 (B) and DiB2-split (E). Super-resolution reconstruction from 5 000 frames of DiB2 (C) and DiB2-split (F); scale bars are 1 µm. The image resolution, as determined by decorrelation analysis, is 31.2 nm and 30.0 nm for panels B and E, respectively.

Supplementary Table S1. Data Collection and Refinement Statistics

	**DiB3 (PDB ID 6UKK)**	**DiB2-split (PDB ID 6UKL)**
Wavelength (Å)	0.97857	0.97857
Space group	C 1 2 1	P 3_2 2 1
Unit cell dimensions	a = 89.6179.<br>b = 38.9929,<br>c = 51.8367,<br>α = 90°,<br>β = 113.208°,<br>γ = 90°	a = b = 68.2405,<br>c = 216.292,<br>$\alpha = \beta$ = 90°,<br>γ = 120°
Resolution range (Å)	30.94 - 1.6 (1.63 - 1.6)	72.10 - 2.02 (2.05 - 2.02)
Total no. of reflections	93,957 (2,730)	346,459 (15,187)
Unique reflections	21,731 (1,012)	39,425 (1,913)
Completeness (%)	98.9 (90.2)	100.0 (97.5)
Multiplicity	4.3 (2.7)	8.8 (7.9)
Mean I / σ(I)	13.4 (1.8)	11.5 (2.1)
$CC_{1/2}$	0.999 (0.899)	0.996 (0.792)
**Refinement Statistics**		
Rwork/Rfree(%)	18.39/19.92	20.51/24.77
Average B factor ($Å^2$)	35.83	39.59
Total no. of atoms	1,359	3,953
Water molecules	81	106
Protein residues	158	457
Bond angles (°)	1.84	1.98
Bond length (Å)	0.015	0.015
Ramachandran: favored/allowed (%)	100/0	98.43/1.57
Clashscore	2.00	5.21
Numbers in parentheses are for the highest-resolution shell.		

Supplementary video 1. Video showing 1 000 frames of HeLa cell transiently transfected with vimentin-DiB2 construct in the presence of 25 nM M739. Plays at 30 fps (acquisition speed 30 Hz, 1.1 kW cm^{-2} of 488 nm laser). Scale bar is 4 μm.

Supplementary video 2. Video showing 1 000 frames of HeLa cell transiently cotransfected with vimentin-splitN$_{1-125}$ + splitC$_{110-177}$-TagBFP constructs in the presence of 25 nM M739. Plays at 30 fps (acquisition speed 30 Hz, 1.1 kW cm^{-2} of 488 nm laser). Scale bar is 4 μm.
